# Feasibility of scaling-up an evidence-based physical activity behaviour change intervention into routine ambulatory hospital care: a retrospective implementation evaluation using the RE-AIM framework

**DOI:** 10.1186/s12889-025-23614-2

**Published:** 2025-07-07

**Authors:** Ashley R. Dunford, Stephen Begg, Michael Kingsley, Paul O’Halloran, Byron M. Perrin, Stephen Barrett

**Affiliations:** 1 Health Promotion Department, Bendigo Health, Bendigo, VIC 3550 Australia; 2 La Trobe Rural Health School, La Trobe University, Bendigo, VIC 3552 Australia; 3 Holsworth Research Initiative, La Trobe University, Bendigo, VIC 3552 Australia; 4 Department of Exercise Sciences, University of Auckland, Auckland, New Zealand; 5 School of Psychology and Public Health, La Trobe University, Melbourne, 3086 Australia; 6 Research and Innovation, Bendigo Health, Bendigo, VIC 3550 Australia; 7 Renal Services, Bendigo Health, Bendigo, VIC 3550 Australia

**Keywords:** Behaviour change, Motivation, Rural health, Health promotion, Chronic disease prevention, Implementation science

## Abstract

**Background:**

Scaling up evidence-based interventions to improve physical activity (PA) is important for enhancing health outcomes. The Healthy4U (H4U) program, initially successful in improving PA and health outcomes among ambulatory hospital patients, was expanded from one regional hospital to five rural hospitals. This study retrospectively examines the feasibility of implementing H4U at Scale (H4U-AS) over 12 months.

**Methods:**

A feasibility implementation evaluation was conducted retrospectively using the RE-AIM (Reach, Effectiveness, Adoption, Implementation, Maintenance) framework. The following variables were assessed within each RE-AIM domain: Reach: Number of program participants. Effectiveness: Measured changes in PA (Metabolic Equivalent of Task minutes (MET-mins/week)), sedentary behaviour (hours/day spent seated), fruit and vegetable intake (serves/day), and nicotine dependence score (Fagerström Test for Nicotine Dependence (FTND)) using paired t-tests or Wilcoxon signed-rank tests. Adoption: Type of setting, program integration, and behaviour change training uptake. Implementation: Participant and hospital recruitment adherence. Maintenance: Continuation of the program.

**Results:**

Reach: In total, 37 participants were recruited during the 6-month recruitment period; pre- and post-data were available for 33. Effectiveness: PA increased from a median of 460 MET-mins/week to 840 (*p* < 0.001). Sedentary behaviour decreased from 8.0 h/day to 7.0 (*p* < 0.001). Vegetable intake increased from 3.0 serves/day to 3.5 (*p* = 0.001). Fruit intake did not change significantly (*p* = 0.228). Nicotine dependence decreased non-significantly from 5.0 to 4.5 (*p* = 0.08). Adoption: The program was successfully implemented in five rural hospitals; feedback from hospital representatives indicated that recruitment procedures were integrated into existing hospital workflows. To support recruitment, processes were adapted to include mailing out invites to people on elective surgery wait lists. Implementation: 86% of participants completed the minimum 4 of 6 available sessions, and all hospitals recruited during the program period. Maintenance: Funding for the project was not available beyond the 12-month period. As a result, recruitment into the program was ceased.

**Conclusion:**

H4U-AS suggests that implementing an evidence-based PA intervention from one regional hospital to five rural hospitals may be feasible. Participants improved PA and dietary behaviours. However, limited participant recruitment during the short recruitment period, and funding cessation, impacted the extent to which the program could be offered and evaluated at scale.

**Supplementary Information:**

The online version contains supplementary material available at 10.1186/s12889-025-23614-2.

## Contributions to the literature

- The findings describe the feasibility of implementing an evidence-based health promotion intervention into rural, real-world settings in a 12-month project.
- This study shows that when rural hospital patients are referred to behaviour change interventions in routine clinical practice, they show similar improvements in physical activity as that found in controlled randomised trials.
- This study applied the RE-AIM framework to examine the scale-up of a physical activity behaviour change intervention and illustrates how the framework can be operationalised in a pragmatic, real-world project.

## Background

Insufficient physical activity (PA) is a major risk factor for chronic diseases [1]. Despite strong evidence of the benefits of regular PA, many adults worldwide do not undertake sufficient PA [2]. Many evidenced based interventions are designed to improve and sustain PA, and scaling up effective interventions is important for widespread impact [3]. Nevertheless, effectively scaling up evidence-based interventions remains a challenge in both well-controlled research settings and in pragmatic, or ‘real-world’ settings [4].

The Healthy4U (H4U) program is an evidence-based behaviour change intervention that emerged as a promising candidate for scaling up. In the original program, hospital surgeons working in ambulatory care clinics co-designed referral pathways that integrated referrals to the H4U behaviour change intervention into routine hospital care [5, 6]. Participants in the H4U trials demonstrated clinically meaningful improvements in physical activity and health outcomes. Specifically, participants increased their moderate-to-vigorous physical activity (MVPA) by 22 min per day at 9-month follow-up, alongside beneficial changes in body mass, waist circumference, and health-related quality of life scores [5, 7–9]. The H4U trials also demonstrated cost-effectiveness and received high levels of participant satisfaction [10, 11]. Participant feedback revealed that surgeon recommendations to increase PA were perceived as both acceptable and influential in initiating behaviour change, and that participants valued retaining autonomy over the decision to engage in follow-on services [11].

Building on these positive outcomes, the H4U program was implemented in five smaller rural hospitals. The regional hospital served as the central coordinating point, providing resources and delivering the intervention [12]. The rural hospitals played a key role in recruiting patients, with input from their representatives to ensure the program was adapted to their contexts. The goal was to expand access to the behaviour change interventions for a larger cohort of rural patients.

The project, known as H4U at Scale (H4U-AS), was designed primarily as a feasibility project, and had an initial commitment of funding for one year only, with potential for further funding. This study describes the feasibility of implementing H4U-AS across the five additional rural hospitals using the RE-AIM (Reach, Effectiveness, Adoption, Implementation and Maintenance) framework.

## Methods

### Study design

This study is a pragmatic evaluation of the feasibility of implementing H4U-AS program using the RE-AIM framework [13]. The RE-AIM framework was applied retrospectively to assess the reach, effectiveness, adoption, implementation, and maintenance of H4U-AS across five rural hospitals in Victoria, Australia.

This is considered a pragmatic evaluation as project funding was provided for service delivery, and not specifically for a research or theoretical perspective. Table 1 outlines how the RE-AIM framework has been applied to measure both participant level and system level outcomes. Data were collected using a combination of quantitative methods (e.g., pre-post measures of participant outcomes) and qualitative methods (e.g., semi-structured interviews with hospital representatives).

**Table 1:** Application of RE-AIM framework to the study

Domain	Description of domain and outcome metric	Outcome measure(s) used	Variables of interest and Tools
Reach	Individual level measure of participation.	Number of participants	Number of participants in the H4U-AS intervention. Recruitment rates based on mail-out invites sent. (Recruitment rates could not be calculated for those recruited from surgical specialist appointments). Collected demographic and participant characteristics.
Effectiveness	Participants’ outcome or benefits received.	Physical activity, sedentary behaviour, diet and nicotine dependence	International Physical Activity Questionnaire (IPAQ) [14], Fagerström Test for Nicotine Dependence (FTND) [15], and serves of fruit and vegetables per day, per the NHMRC’s Australian Dietary Guidelines [16].
Adoption	Setting/location program was adopted.	Type of setting, involvement in recruitment and integration of the program.	Number and type of settings (e.g., hospital, local health service) and feedback on integration into routine care.
Implementation	Factors related to the implementation of the program.	Participant adherence. Health service adherence in offering the program.	Measured adherence by program completion (attending 4 of 6 sessions). Regular contact with hospital representatives to monitor adherence.
Maintenance	Whether the program is maintained (or sustained) post implementation.	Number of sites that offered H4U-AS post-funding cessation.	Number of sites offering H4U-AS post-funding. No participant related maintenance data due to lack of long-term follow-up.

*H4U-AS* Healthy 4U at scale, *MI-CBT* motivational interviewing and cognitive behavioural therapy

### The healthy 4U program

The original H4U program has been running for seven years in a large tertiary hospital in regional Victoria, Australia. The program has demonstrated effectiveness in promoting PA and improving health outcomes through two randomised controlled trials [5, 8] and two economic analyses [7, 10].

A key strength of the H4U program was its co-designed recruitment process, developed in collaboration with the hospital surgeons [5, 6]. Collaborating surgeons represented the specialties of general surgery and orthopaedics, with referred patients undergoing elective procedures such as hernia repair, cholecystectomy, gastrointestinal surgeries and joint replacements. The surgeons worked with the H4U team to create tailored recruitment materials and engaged patients in brief conversations about increasing PA. Surgeons then provided patients with a recruitment flyer, seamlessly integrating preventive practices into routine outpatient care. In one trial, approximately 20% of the 2076 patients who received flyers enrolled in the program [5].

### Healthy 4U at scale

**Fig. 1:**
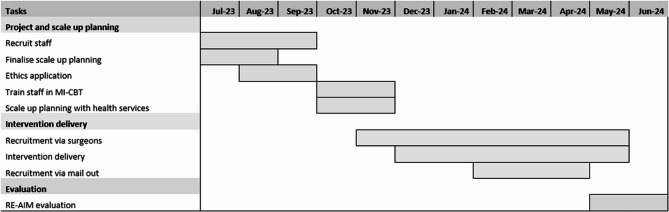
Healthy 4U 12-month project timeline of major tasks

In July 2023, the Victorian Department of Health’s Planned Surgery Recovery and Reform program provided funding to support behaviour change pathways for individuals preparing for surgery. Funding was awarded to the Loddon Mallee Health Network, an unincorporated joint venture governed by CEOs of public hospitals from the Loddon Mallee region [17] to implement the H4U program into 5 additional rural hospitals that provided elective surgery. The rural hospitals were selected by the Planned Surgery Recovery and Reform coordinator based on existing partnerships within the Loddon Mallee Health Network, where surgeons from the coordinating hospital provided outreach clinics and had established referral pathways. A part-time project supervisor (0.1 FTE) and project officer (0.4 FTE) were employed to run the H4U-AS project. The 12-month project timeline is provided in Fig. 1.

Participants in H4U-AS were offered six 20-minute telephone-delivered sessions of motivational interviewing and cognitive behaviour therapy (MI-CBT) over 12 weeks. In line with the original H4U trials, where intervention adherence rates were 96% (5 sessions) and 100% (8 sessions) respectively, program completion for H4U-AS was pragmatically defined a priori as attending at least 4 of the 6 offered sessions (≥ 80% attendance). The intervention components are detailed in Additional file 1. An Accredited Practising Dietitian was recruited in September 2023 to deliver the MI-CBT intervention. The clinician was trained in MI-CBT, including workshop attendances with an experienced practising Sports and Exercise Psychologist; this trainer is also a member of Motivational Interviewing Network of Trainers. The clinician also received one-on-one coaching from the clinician who delivered the MI-CBT interventions in the H4U trials. Proficiency was confirmed using the MI-CBT fidelity scale during role play sessions [18].

Five rural hospitals that provide elective surgery were chosen to be part of the scale up. Between September and November, the project supervisor engaged with representatives from the 5 hospitals and discussed the implementation and scale up, facilitated by the use of the PRACTIS (Practical Planning and Assessment to Facilitate Translation of Interventions in Public Health Practice) guide (Table 2) [19]. The hospital representatives collaborated with their respective surgical teams; across all five sites the preferred method was equipping surgeons with referral material and detailed information about the service. Recruitment flyers and posters were distributed to the rural hospitals in November, and recruitment began the same month.

**Table 2:** Application of PRACTIS guide in the implementation of the H4U program at scale

Key PRACTIS Component	Planning and Implementation Details
Stakeholder Engagement	• Engaged stakeholders from Mildura Base Public Hospital, Swan Hill District Health, Kerang District Health, Echuca Regional Health, and Dhelkaya Health • Stakeholders requested to engage with included surgeons, program coordinators, administrative staff • Conducted individual interviews to gather input • Stakeholder input shaped the integration of referrals into outpatient surgical care, ensuring alignment with existing workflows and patient needs
Contextual Adaptation	• Ensured recruitment strategies to fit the local contexts of participating health services • Flexible approach to align with the preferences at each site
Implementation Planning	• Developed clear implementation plans detailing roles and responsibilities of health service representatives • Ensured coordination and clarity across different sites • Established timelines and milestones for program rollout
Capacity Building	• Provided training sessions for clinician delivering the MI-CBT behaviour change intervention • Emphasised proficiency in the use of MI-CBT through treatment manuals and fidelity anchor point scales
Evaluation Design	• Designed evaluation methods to be pragmatic and feasible • Employed both quantitative and qualitative measures • Metrics included reach, effectiveness, adoption, implementation, and maintenance of the program • Developed tools for data collection including semi-structured interviews

In January 2024, after consulting with the 5 rural hospitals an addendum recruitment procedure was introduced. Patients on elective surgical waitlists whose postcodes matched the geographic areas of the participating hospitals were mailed information regarding the H4U-AS program, and invited to participate in the telephone coaching while on the elective surgery waiting list. In total, 2,204 invitations were mailed out between February and April 2024.

In June 2024, the Victorian Government in Australia cut funding to the public health system, requiring hospital administrations to cut annual operating budgets. Programs funded by Victorian Department of Health’s Planned Surgery Recovery and Reform program, including the H4U-AS program were among those that did not receive ongoing funding. As a result, the project team could no longer deliver the intervention and recruitment into the program ceased in May 2024.

### Data collection

Data on patients’ demographics, outcomes and reasons for participation or barriers to enrolment were collected by the H4U-AS project worker. The H4U-AS project supervisor conducted semi-structured telephone interviews with the hospital representatives after completion of the H4U-AS program to obtain feedback about the program from each hospital’s perspective. The interview guide was specifically developed for this study and is available in Additional file 2. The variables of interest and tools used can be found in Table 1.

### Data analysis

Data were analysed using R (v4.4.0). The Shapiro-Wilk test was used to assess the distribution of variables in the ‘effectiveness’ domain. Paired t-tests were used for normally distributed data, while the Wilcoxon signed-rank test was applied to non-normally distributed data. For key outcomes, 95% confidence intervals (CIs) were calculated to show the range of uncertainty. *P*-values of less than or equal to 0.05 were considered statistically significant. For each of the other RE-AIM domains, a content analysis was conducted to identify key themes from the qualitative feedback obtained from hospital representative interviews. Descriptive statistics were used to summarise the rate of recruitment, adherence, number of settings, and the number of sites maintaining the program after funding ended.

## Results

### Reach

Between December 2023 and May 2024, there were 68 unique enquiries from potential participants and 37 individuals participated in the H4U-AS program. The baseline characteristics of the 37 participants are shown in Table 3. Participants were predominantly female (59%), with a mean age of 55 years. Participants represented 6 of the 10 local government areas in the Loddon Mallee region, and most were scheduled for general or gynaecological surgery. Most selected physical activity as their focus area. In total, 3 individuals were recruited from surgeon pathways, and 34 recruited from the mail out process. The recruitment rate from surgeons providing flyers to patients was estimated to be 0.2% based on 1,500 flyers being provided to clinics across all sites, and the recruitment rate from the addendum the mail out process was 1.5%.

**Table 3:** Baseline characteristics of participants

Variable	
Participants	37
Age (years)	55 (± 8)
Sex: female, n (%)	22 (59%)
Aboriginal or Torres Strait Islander	2 (5%)
Local Government Areas	
Swan Hill	6
Campaspe	11
Gannawarra	6
Mitchell	5
Mildura	2
Mount Alexander	7
Surgical speciality	
Orthopaedic	5
General surgery	15
Ophthalmology	2
Gynaecology	10
Gastro surgery	4
ENT Surgery	1
Coaching timeframe	
Pre-surgery	23
Had surgery during telephone coaching period	4
Post-Surgery	10
Focus area for intervention	
Physical activity	24
Smoking	2
Diet	3
Multiple behaviours	11

Of the 31 individuals who inquired about the program but did not enrol, the main barriers to enrolment were self-reported challenges in ambulatory status, which they indicated affected their ability to make PA changes.

### Effectiveness

For participant level outcomes, pre- and post-intervention data were available for 33 participants. The participants increased PA from baseline to follow-up, where median MET-minutes/week increased from 460 (95% CI:169, 480) pre-intervention to 840 (95% CI: 600, 989) post-intervention (*p* < 0.001). This represents a change from 115 min of moderate to vigorous physical activity (MVPA)/week (16.5 min/day) to 210 min of MVPA/week (30 min/day), with 4 METs commonly used as a representative value for moderate-intensity exercise, as per IPAQ scoring guidelines [17]. Sedentary behaviour (hours spent seated per day) decreased significantly from a median of 8.0 h (95% CI: 7.4, 8.6) to 7.0 h (95% CI: 6.4, 8.0) (*p* < 0.001). Serves of vegetables per day increased significantly from a median of 3.0 serves per day (95% CI: 3.0, 4.0) to 3.5 serves per day (95% CI: 3.2, 4.0) (*p* = 0.001). Serves of fruit per day did not change significantly, remaining at a median of 1.0 serve per day (95% CI: 0.7, 1.5 pre-intervention and 95% CI: 1.0, 2.0 post-intervention) (*p* = 0.228). For participants that smoked, nicotine dependence declined non-significantly from a mean of 5.0 (95% CI: 4.0, 6.5) pre-intervention to 4.5 (95% CI: 3.0, 6.5) post-intervention (*p* = 0.08).

### Adoption

Five rural Victorian hospitals implemented the H4U-AS program. This included one small health service (< 50 acute care beds) and four medium health services (> 50 acute care beds). Each of these hospitals offer outpatient clinics catering to surgical stream patient consultations, which eliminated the need for separate contextual process adaptations. This uniformity facilitated a consistent approach to program integration across the 5 sites. Feedback from hospital representatives indicated that the surgeons were able to integrate referrals to H4U-AS into existing hospital workflows.

Challenges with recruitment were discussed with hospital representatives during regular scheduled meetings. During these meetings the idea of using the elective surgery waitlist as a source for recruitment was discussed by some hospital representatives and this pathway was adopted into the implementation process.

### Implementation

Adherence data were available for all 37 participants, with 33 participants (approximately 86%) completing 4 or more sessions. In all the participating hospitals the implementation planning process was facilitated by a hospital representative, this included directors of surgical services, director of nursing and nurse unit managers. Regular contact was maintained with the hospital representatives over the project duration via regular scheduled meetings. None of the surgeons consulting in the hospitals and recruiting for the H4U-AS program were spoken to directly by the project team. Time constraints and competing priorities were the biggest barriers to the surgeon’s capacity to engage with the H4U-AS project team.

Flyers and posters were widely circulated to surgical staff and consulting rooms, and information was placed on computer screensavers in one hospital as a reminder. Health service representative feedback indicated that the surgeons supported the program, were providing their patients with the recruitment material, and were able to integrate referrals into clinical care. However, the project team did not have direct contact with the surgeons, which may have limited the recruitment rates compared to the original H4U program, where direct engagement with surgeons led to higher participation.

### Maintenance

No hospital stopped participation in the H4U-AS program during the funding period. All hospitals, participating in regular scheduled meetings with the project team, reported a willingness to continue with the program beyond the initial 12-month period, however, due to Victorian Government funding cuts, recruitment into the program was unable to be maintained.

## Discussion

Scaling up and implementing evidence-based health interventions in rural healthcare settings represents a critical yet complex goal, requiring careful consideration of program adaptation, stakeholder engagement, and sustainability [4, 19]. This study retrospectively examined the implementation of the H4U-AS program across five rural hospitals, evaluating its effectiveness while addressing key challenges related to adoption and implementation.

In total, 37 participants were recruited during the 6-month recruitment window, with 33 participants demonstrating strong engagement by completing 4 or more of the 6 available sessions. Participants showed improvements in PA and sedentary behaviour, consistent with previous findings of MI-CBT for PA change in clinical trials [5, 8]. The reported increase of 17 min of MVPA per day is important, as 15 min per day of MVPA can decrease chronic disease risk [20] and reduce all-cause mortality [21]. Improvements were also reported for vegetable intake and for nicotine dependence, supporting the growing evidence that MI-CBT interventions can effectively promote lifestyle behaviour change [22, 23]. Future randomised trials should further explore the effect of MI-CBT on dietary intake and nicotine dependence, which could provide valuable insights into the intervention’s efficacy across multiple health behaviours.

The use of the PRACTIS guide facilitated stakeholder engagement and permitted context-specific adaptations at each hospital. By employing prospective planning guided by a structured framework, the initiative not only streamlined implementation but also minimised the time required for adjustments and decision-making at each site, ensuring a more efficient and coordinated approach to achieving the implementation goals. This structured planning allowed surgeons to refer patients to the intervention in routine ambulatory care, although the lack of direct engagement with surgeons may have limited the program’s reach. This limitation was particularly evident when comparing the original H4U program [5], delivered at a single site, to the hub-and-spoke approach used in H4U-AS. In H4U-AS all contact was with hospital representatives rather than directly speaking to patient-facing surgeons. Without direct engagement, it was not possible to fully understand surgeons’ perceptions of the program or how it aligned with their practices and beliefs, potentially leading to the very low recruitment rates from surgeon interactions. This contrasts to our previous studies where direct engagement with surgeons enabled the co-design of referral pathways, which supported patient engagement and led to a recruitment rate of approximately 20% from surgeon interactions [5]. Involving stakeholders and fostering a sense of ownership can significantly enhance the adoption and integration of programs into routine care [19, 24]. Although the PRACTIS guide informed the implementation, the limited direct involvement of surgeons in H4U-AS likely hindered program reach, as successful health promotion interventions often depend on strong networks, relationships, and shared priorities [25–27]. Moving forward, it is crucial to involve all key stakeholders directly, ensuring their buy-in and alignment with program goals to enhance recruitment and program uptake [28, 29].

The primary aim of this project was to explore the feasibility of implementing this intervention across five additional hospitals, and not how to sustain delivery long-term. Interest in longer-term maintenance was expressed, and all hospitals were willing to maintain these referral pathways beyond the original planned 12-months. However, broader health system pressures prevented continuation. In mid-2024, significant healthcare budget cuts were announced across Victoria, requiring health services to achieve financial breakeven by June 2025 through cost-containment measures such as redundancies of frontline staff and reductions in services. As a result, funding for many elective surgery reform programs were cut, and funding for project staff was withdrawn, preventing further delivery of the intervention. None of the rural hospital staff were trained to deliver the intervention independently. This highlights the vulnerability of preventative health initiatives to external funding and policy changes, particularly in resource-constrained rural settings. Longer-term funding models that include internal capacity building, such as training hospital staff to deliver interventions, could enhance sustainability and resilience to systemic shocks.

Integrating behaviour change interventions into routine surgical and hospital care is increasingly recognised as a strategy to improve patient outcomes and system efficiency, but requires deliberate planning, resourcing, and system-wide alignment [5, 30]. A recent national survey of NHS hospitals in the UK found that many orthopaedic units have embedded structured prehabilitation and pre-operative education into routine care for joint replacement patients, supported by dedicated teams, standardised referral processes, and clinical governance structures [31]. Broader models have also been described to guide the establishment of such programs [32]. While these structured prehabilitation and pre-operative education models differ in intent and scope from H4U-AS, they offer a valuable example of how preventive health activities can transition from project-based initiatives to integrated, policy-supported elements of care delivery.

Building such integration through training and workflow redesign may help programs like H4U-AS become sustainable components of routine care. Furthermore, framing preventive interventions as both clinically relevant and cost-efficient may assist with advocating for ongoing investment and policy support [7, 33]. Future implementation research should explore how behaviour change interventions can be aligned with existing care structures and sustained through internal resourcing and system-level commitment.

### Strengths and limitations

Evaluations of scaled-up delivery of evidence-informed interventions are scarce, and even more so in real world, non-research settings. This evaluation of a pragmatic, rapid implementation project offers valuable insights into successes and challenges of real-world implementation. Notably, over 90% of participants were recruited from elective surgery waitlists, and this cohort demonstrated significant improvements in physical activity and sedentary behaviour. This suggests that elective surgery waitlists represent a large and accessible population that may benefit from behaviour change interventions, and this approach could be a low-cost strategy to promote health improvements in this group.

For participants, the MI-CBT intervention demonstrated significant improvements in PA and reductions in sedentary behaviour, supporting its potential for broader application in lifestyle behaviour change. The MI-CBT intervention was delivered following comprehensive training and the use of fidelity scale anchor points during training sessions [18]. While we did not assess fidelity during the intervention sessions, the successful delivery of the program by the trained facilitator demonstrates that with adequate training, health professionals can implement MI-CBT interventions. The design of future MI-CBT interventions to promote changes in PA, diet and smoking may be supported by development of consensus on key intervention components [34].

There are some limitations to this study. First, participant outcomes were examined using pre-post methods and there was no randomisation of participants. Without a control group, it is difficult to attribute the observed changes solely to the intervention. However, the trajectory of change in PA outcomes observed here aligns with those seen in the H4U randomised trials, where intervention groups showed marked improvements, while control groups experienced either minimal or no change in PA outcomes [5, 8]. Second, outcome measures were based on self-reported data, introducing potential biases such as recall or social desirability bias. Third, the RE-AIM framework was applied retrospectively, which may introduce bias; however, its application provided a structured approach to assess key implementation outcomes across multiple domains. Fourth, the need to introduce an additional recruitment method midway through the project highlights a potential limitation to scalability. Fifth, the use of validated questionnaires strengthens the reliability of these measures but does not entirely eliminate bias. Lastly, given the small sample size and the unique characteristics of rural hospital settings, caution is warranted when generalising these findings to other contexts.

## Conclusion

The evaluation of the H4U-AS program demonstrates that implementing an evidence-informed behaviour change intervention from one hospital to five additional hospitals may be feasible, with participants showing improvements in health behaviours. Rural hospitals engaged in implementation planning and adopted the recruitment processes for their respective sites. In contrast to previous work, the project team had no direct engagement with the patient-facing clinicians which may have had some impact on the reach of the program. Although all hospitals were willing to continue the program, government funding was not continued after 12-months, which resulted in cessation of the service.

## Supplementary Information

Supplementary Material 1.

## Data Availability

The datasets used and/or analysed during the current study are not openly available due to reasons of sensitivity and are available from the corresponding author upon reasonable request. Data are located in controlled access data storage at Bendigo Health.

## Abbreviations

FTE: Full time equivalent
FTND: Fagerström Test for Nicotine Dependence
H4U: Healthy 4U
H4U-AS: Healthy 4U at Scale
IPAQ: International Physical Activity Questionnaire
MET: Metabolic Equivalent of Task
MI-CBT: Motivational Interviewing and Cognitive Behavioural Therapy
MVPA: Moderate-to-Vigorous Physical Activity
PA: Physical Activity
PRACTIS: Practical Planning and Assessment to Facilitate Translation of Interventions in Public Health Practice
RE-AIM: Reach, Effectiveness, Adoption, Implementation and Maintenance
