# Supplementary material for: Feasibility of scaling-up an evidence-based physical activity behaviour change intervention into routine ambulatory hospital care: a retrospective implementation evaluation using the RE-AIM framework
Source: BMC Public Health. 2025 Jul 7;25:2396. doi: 10.1186/s12889-025-23614-2 (PMC12232822; doi:10.1186/s12889-025-23614-2)
Supplement: Supplementary file 1 — Supplementary Material 1. [file 12889_2025_23614_MOESM1_ESM.docx]

**Feasibility of scaling-up an evidence-based physical activity behaviour change intervention into routine ambulatory hospital care: a retrospective implementation evaluation using the RE-AIM framework**

**Additional Files**

**Additional File 1: Detailed Intervention Description Using TiDIER Framework**

**1. Brief Name**

MI-CBT behaviour change intervention for Health Behaviour Modification (Healthy4U Program)

**2. Why**

The Healthy4U Program aims to improve physical activity levels, dietary habits, and smoking cessation among patients preparing for surgery, based on evidence linking these factors to better postoperative outcomes.

**3. What (Materials)**

- Informational posters and flyers for clinics
- Telephone coaching scripts integrating Motivational Interviewing (MI) and Cognitive Behavioural Therapy (CBT)
- Participant experience surveys

**4. What (Procedures)**

Participants received up to six 20-minute telephone coaching sessions over 12 weeks. These sessions utilised MI micro-skills and CBT strategies to address physical activity, diet, and smoking behaviours. The coaching focused on open-ended questions, affirmations, reflections, summaries, and techniques to enhance motivation, self-efficacy, and behaviour change.

**5. Who Provided**

The intervention was delivered by a Dietitians Association Accredited Practising Dietitian trained in MI-CBT techniques.

**6. How**

The intervention was delivered via telephone, allowing participants to receive coaching from the comfort of their homes.

**7. Where**

The intervention targeted rural patients from five health services within the Loddon Mallee Health Network in rural Victoria, Australia.

**8. When and How Much**

Participants were offered up to six coaching sessions over a 12-week period. Each session lasted approximately 20 minutes.

**9. Tailoring**

The program was adapted to fit the local context of each participating health service. Recruitment strategies were flexible to align with the preferences and logistical realities of each site as suggested by the stakeholders.

**10. Modifications**

To address low engagement rates, the recruitment strategy was modified to include a mail-out process. Information about the telephone coaching was sent to patients on Bendigo Health’s planned surgery waitlist in postcodes outside of greater Bendigo, corresponding to the rural health services.

**11. How Well (Planned)**

PRACTIS guide informed planning the scaling of the intervention. Fidelity to the intervention was assessed during training only, using MI-CBT manuals and fidelity scales**.**

**12. How Well (Actual)**

Intervention proficiency was achieved; however, engagement rates were below expectations. Qualitative feedback from participants and staff helped identify areas for improvement.

**Additional File 2: H4U-AS interview guide**

**Overall Perception of the Project**

- From your perspective, how would you describe the overall process of being involved in the telephone coaching project?

**Reach (Who Participated and Who Didn’t?)**

- How well do you think the program reached the intended group - people on elective surgery waitlists?
- What do you think were the biggest barriers to patient recruitment?
- What strategies, if any, did your hospital use to help with recruitment?

**Effectiveness (Impact on Patient Outcomes)**

- Based on what you observed or heard, how did the program impact patient preparedness for surgery?
- Did you notice any indirect benefits, such as changes in patient engagement with hospital services or staff interactions?

**Adoption (Integration into Hospital Systems)**

- How was the program communicated and introduced to hospital staff, and particularly surgeons?
- What level of engagement did your surgeons have with the program?
- What level of engagement did your hospital’s leadership with the program?
- Were there any concerns raised by hospital staff about the program?

**Implementation (What Worked and What Didn’t?)**

- What were the biggest challenges your hospital faced in supporting this program?
- Were there any logistical or administrative difficulties in referring patients?
- What worked well in coordinating the program between hospitals and the telephone coaching service?
- What additional support or resources would have made implementation easier?

**Maintenance (Sustainability and Future Directions)**

- Do you see a long-term role for this program within your hospital? Why or why not?
- What changes would need to be made for the program to be more sustainable?
- If additional funding were available, how could this program be improved or expanded?
